# The Use of Digital Pathology and Artificial Intelligence in Histopathological Diagnostic Assessment of Prostate Cancer: A Survey of Prostate Cancer UK Supporters

**DOI:** 10.3390/diagnostics12051225

**Published:** 2022-05-13

**Authors:** Kai Rakovic, Richard Colling, Lisa Browning, Monica Dolton, Margaret R. Horton, Andrew Protheroe, Alastair D. Lamb, Richard J. Bryant, Richard Scheffer, James Crofts, Ewart Stanislaus, Clare Verrill

**Affiliations:** 1 Institute of Cancer Sciences, University of Glasgow, Switchback Road, Glasgow G61 1QH, UK; 2 Department of Pathology, Queen Elizabeth University Hospital, Govan Road, Glasgow G51 4TF, UK; 3 Department of Cellular Pathology, Oxford University Hospitals NHS Foundation Trust, John Radcliffe Hospital, Headley Way, Oxford OX3 9DU, UK; richard.colling@pmb.ox.ac.uk (R.C.); lisa.browning@ouh.nhs.uk (L.B.); clare.verrill@ouh.nhs.uk (C.V.); 4 Nuffield Department of Surgical Sciences, University of Oxford, John Radcliffe Hospital, Headley Way, Oxford OX3 9DU, UK; monica.dolton@nds.ox.ac.uk (M.D.); alastair.lamb@nds.ox.ac.uk (A.D.L.); richard.bryant@nds.ox.ac.uk (R.J.B.); richard.scheffer@btinternet.com (R.S.); croftsj@hotmail.com (J.C.); ewartbs@hotmail.com (E.S.); 5 NIHR Oxford Biomedical Research Centre, Oxford University Hospitals NHS Foundation Trust, John Radcliffe Hospital, Headley Way, Oxford OX3 9DU, UK; 6 Paige AI, 11 Times Sq, Fl 37, New York, NY 10036, USA; margaret.horton@paige.ai; 7 Department of Oncology, University of Oxford, Roosevelt Drive, Oxford OX3 7DQ, UK; andrew.protheroe@oncology.ox.ac.uk; 8 Oxford Cancer & Haematology Centre, Oxford University Hospitals NHS Foundation Trust, Churchill Hospital, Old Road, Oxford OX3 7LE, UK; 9 Department of Urology, Oxford University Hospitals NHS Foundation Trust, Churchill Hospital, Old Road, Oxford OX3 7LE, UK

**Keywords:** prostate cancer, digital pathology, artificial intelligence

## Abstract

There has been particular interest in the deployment of digital pathology (DP) and artificial intelligence (AI) in the diagnosis of prostate cancer, but little is known about the views of the public on their use. Prostate Cancer UK supporters were invited to an online survey which included quantitative and qualitative questions exploring views on the use of DP and AI in histopathological assessment. A total of 1276 responses to the survey were analysed (response rate 12.5%). Most respondents were supportive of DP (87%, 1113/1276) and of testing AI in clinical practice as a diagnostic adjunct (83%, 1058/1276). Respondents saw DP as potentially increasing workflow efficiency, facilitating research, education/training and fostering clinical discussions between clinician and patient. Some respondents raised concerns regarding data security, reliability and the need for human oversight. Among those who were unsure about AI, information was requested regarding its performance and others wanted to defer the decision to use it to an expert. Although most are in favour of its use, some are unsure, and their concerns could be addressed with more information or better communication. A small minority (<1%) are not in favour of the testing of the use of AI in histopathology for reasons which are not easily addressed.

## 1. Introduction

The role of the histopathologist (cellular pathologist) in clinical care is poorly understood by patients and the wider public [1] despite the histopathology contribution being integral for the diagnosis and management of conditions such as cancer. The practice of histopathology has rapidly evolved over the last 10 years, with now well-established advances in molecular diagnostics and more recently the introduction of digital pathology (DP). With the impending roll out of artificial intelligence (AI), it is important to understand what level of detail the public wish to know about these advances, the role they play in diagnostic processes, what level of acceptance of such changes is likely (and why) and how we communicate these advances.

The basic techniques used in histopathology emerged in the 19th century and have not fundamentally changed to the present day [2]. They centre on the examination of stained sections of formalin-fixed paraffin-embedded tissue using light microscopy. Over the last 50 years, however, there has been an exponential growth in the complexity of tumour classification with the advent of immunohistochemistry and, in recent decades, the integration of genetic and molecular methods into diagnostics. There have been further advances in the last decade with the adoption of DP. With DP, the glass slide is scanned and the digital whole slide image is examined on a computer screen instead of a microscope. DP is gaining traction as a primary reporting modality in many cellular pathology centres in the United Kingdom (UK) [3] and is likely to become mainstream in the histopathology workflow in the coming years [4]. A survey conducted in 2017 [5] reported that 60% of pathology centres in the UK had access to DP equipment and 58.5% considered the development of DP infrastructure to be a priority.

The adoption of DP may bring improvements to the diagnostic workflow, allowing streamlined prioritisation, greater flexibility in remote working, potential financial savings [6] and ease of research collaboration [7]. Education of histopathology trainees traditionally relies on face-to-face case review with a consultant trainer on a multi-header microscope. DP allows these interactions to take place remotely, facilitating training across multiple geographical sites [8]. These benefits have particularly been demonstrated in recent years due to the COVID-19 pandemic [3]. The majority of histopathology trainees polled in a 2019 survey saw the introduction of DP into their training as a positive step [9]. Furthermore, there is potential to develop supraregional networks, streamlining the process where certain cases require external review [10].

Digitisation of histopathological images brings the potential for implementation of novel diagnostic adjuncts such as AI-assisted diagnostics. These may provide diagnostic assistance to histopathologists in assessing current standard of care features and may provide novel insights into disease biology not otherwise possible with a human observer. Examples include automation of tasks such as quantification of mitotic index [11,12] or hormone receptor scores in breast cancer [11]. Furthermore, there is evidence emerging of the potential application of deep learning in AI-assisted diagnostics to gain novel insights into morpho-molecular associations. For instance, the ability to predict aspects of the molecular profiles of malignant tumours, such as EGFR in non-small cell lung [13] cancer and microsatellite instability in gastrointestinal cancers [14], from whole slide images has been demonstrated.

One area of pathology where there has been intense interest in AI is in prostate cancer (PCa). There is potential for improved diagnostic accuracy, improved efficiency and standardisation of prognostic features such as Gleason grading and tumour quantification. The mainstay of diagnosis in PCa is core biopsy and the determination of the grade is by assessment of the morphological features by a histopathologist. These features are then assigned a grade according to the Gleason grading system (Grade Group System), which remains pivotal in prognostication and influences downstream patient management. In cases where the morphological features fall short of a definitive diagnosis of malignancy, such as in limited foci of potential tumour, ancillary studies, including immunohistochemistry to demonstrate loss of basal markers, are requested to aid the reporting histopathologist in their decision. Specific AI applications exist as adjuncts to the reporting of prostate core biopsies, such as suggestion of diagnosis and attribution of Gleason Score [15] and the pre-emptive requesting of immunohistochemistry in morphologically equivocal cases [16]. Some of these algorithms have been cleared with CE marking or FDA clearance for in vitro diagnostics and these are the first such regulatory clearances in the field [15,17]. With approximately 45,000 new diagnoses of PCa per annum in England and Wales, the benefits to the workflow are considerable [18].

The potential to use AI in histopathology practice is a potential paradigm shift in the way histopathologists work and marks a significant step forward beyond traditional light microscopy; however, the adoption of this is currently occasionally being used in specific diagnostic settings [19]. While we seek to accelerate adoption and unlock benefits of this technology, we need to be mindful of any public scepticism and concern regarding the use of technology and AI in modern life, including in healthcare. Although not specific to healthcare, a large-scale international survey of over 150,000 respondents demonstrated mixed opinion regarding implementation of AI in decision making [20]. Differences in acceptance were observed among individuals from certain demographic groups. Higher rates of acceptance were observed in those residing in Asian countries compared to western countries, and in executives and professionals versus manual and service workers.

Few studies exist exploring public perceptions about the deployment of these technological advances to patient care and there are none specific to histopathology. The view towards AI in medicine more broadly appears somewhat split. Public comments on social media in China [21] include broad support for AI in healthcare with many comments suggesting AI could replace doctors entirely. Women undergoing breast screening mammography in Scotland were found to be supportive of AI as an adjunct to diagnosis but were sceptical of fully autonomous AI [22].

Our study aims to analyse the views of a group of the public, specifically supporters of a UK-based prostate cancer charity who have previously undergone prostate biopsy. Our particular objectives were: (1) to explore the current understanding of how biopsies are reported; (2) to assess views on digitisation of histopathology; and (3) to determine whether patients would support the use of AI-assisted diagnostics as an adjunct to the routine reporting of prostate biopsies and histopathological specimens more broadly.

## 2. Materials and Methods

### 2.1. Survey Design

This study collected anonymous, non-NHS data which could not be traced back to an individual. The questions had been developed with the health information team at Prostate Cancer UK (PCUK). Given the possibility that the target population may have had limited familiarity with histopathology, questions were carefully worded to ensure comprehension. The survey was drafted with the biopsy pathway in mind, with internal review to ensure it met the needs of the study along with the needs of the charity and its users. The survey included 11 questions, and follow up questions enabled respondents to make further free text comments. The survey was developed following discussions with a small number of volunteer PCUK supporters. The survey was circulated to PCUK supporters through a mailing list for newsletters etc maintained by PCUK, focussing on men > 50 years old as this is the group who were most likely to meet the inclusion criteria (see below). The online survey was hosted on the Toluna platform (an end-to-end consumer intelligence platform where surveys can be designed and distributed to PCUK supporters); no personal data was collected, and individual respondents could not be traced. The survey was circulated on 21 May 2021 and closed in June 2021. In total, 1307 responses were obtained following circulation to 10,465 PCUK supporters (12.5% response rate).

### 2.2. Survey Composition

The survey is included as supplementary material. Briefly, limited demographic data were available (country of the respondent and, if UK-based, the region—divided into North and Scotland/Northern Ireland/Midlands and Wales/South. Respondents were questioned regarding their baseline knowledge of histopathology and the role of histopathologists in PCa diagnosis. As histopathology services adopt DP and look towards AI for diagnostic support, respondents’ attitudes to the use of these technologies were explored. We specifically explored whether respondents would consider it useful to be able to view digital images of their prostate biopsy enabled by digital pathology, to aid understanding. We explored support for AI, and where there was uncertainty or negativity, we sought to explore the qualitative comments to understand these hesitations.

The survey included an introductory statement (please see online supplementary material). Completion of the survey was taken as consent to participate. There was an explicit statement about publication in an academic journal.

The only inclusion criterion was having had a prostate biopsy at any point (please see online supplementary material, Question S1). Not having had a prostate biopsy, or being unsure, was an exclusion criterion as we were aiming to capture experiences of those who had had a prostate biopsy which required histopathological assessment. A response of “no” or “did not know” to a prostate biopsy in the past resulted in the end of the survey.

### 2.3. Statistical Analysis

Descriptive statistics with graphical outputs were used to characterise survey results. Responses for free text questions were analysed, pertinent themes extracted and categorised according to said themes. Ambiguous or unclear comments were not included.

## 3. Results

### 3.1. Response

In total, 1307 responses were received, of which 87.2% were UK-based; 1276 men responded ‘yes’ to a history of previous prostate biopsy. The remaining respondents were excluded from further questioning.

### 3.2. Understanding of the Role of Histopathology

**Figure 1:**
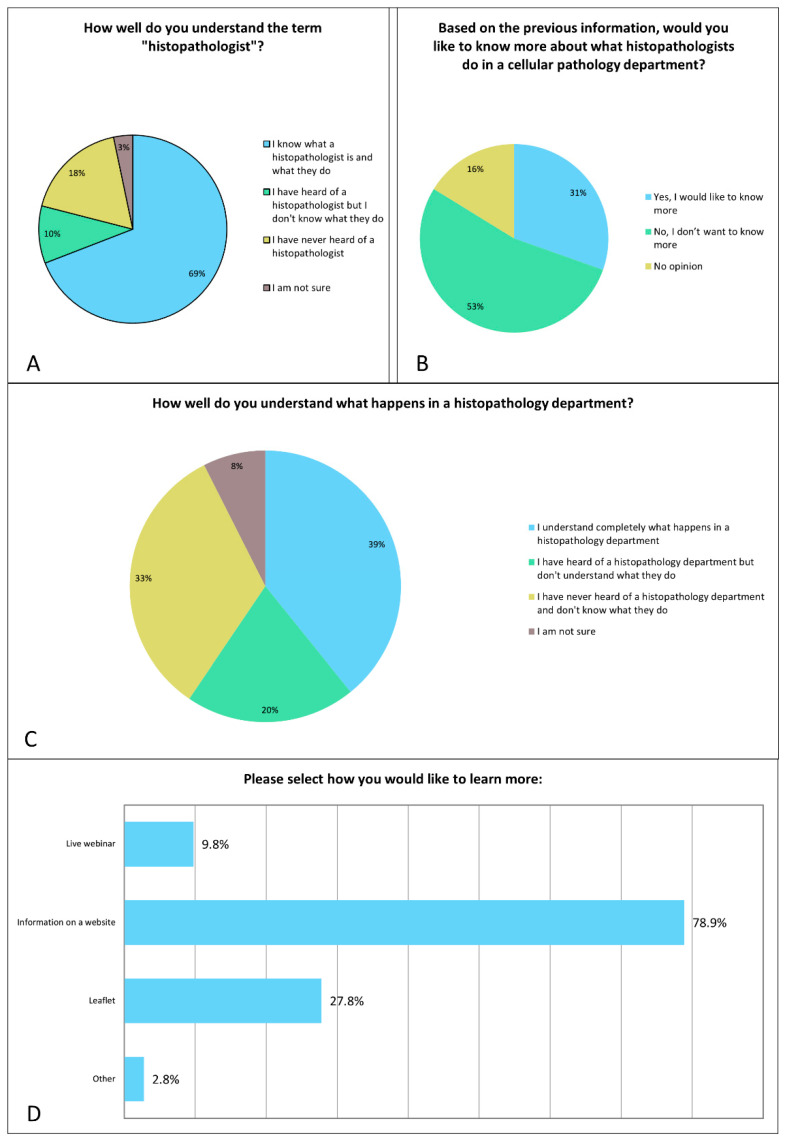
Breakdown of responses on patient understanding of histopathology (**A**–**C**). Those who indicated they wished to know more (388 respondents) shown in (**D**).

The majority (69.1%, 882/1276) stated that they understood the role of the histopathologist (please see online supplementary material, Questions S3–S6, and Figure 1). A further 10% (126/1276) stated they were aware of the term but unclear about the exact nature of the work carried out by histopathologists. A small proportion (39.2%, 500/1276) said that they understand the overall role of a histopathology department.

When asked whether they would wish to know more about the work of histopathologists or of cellular pathology departments, a third responded positively (30.4%, 388/1276), with a small number stating they had no opinion (16.2%, 207/1276). Around half stated they did not wish to know more (53.4%, 681/1276).

### 3.3. Views on Digitisation of Pathology

**Figure 2:**
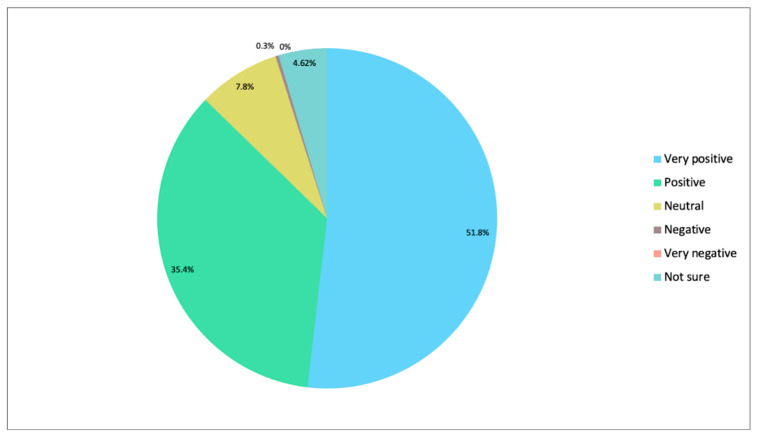
Responses to the question “Some histopathology departments are now going ‘digital’. Slides containing prostate biopsy tissue can now be scanned and viewed digitally on a screen rather than through a microscope. This makes a permanent digital record of the biopsy which reduces the chances of any issues with viewing slides. This also allows histopathologists to easily get a second opinion on a diagnosis. Do you see this change in diagnosing prostate cancer as a positive or negative?” (Please see online supplementary material, Question S7).

Most men viewed the digitisation of histopathological slides as either positive or very positive (87.2%, 1113/1276; please see online supplementary material, Question S7, and Figure 2 and Table 1). Review of free text responses revealed five broad themes: a perception of increased efficiency of turnaround time, record permanence, ease of sharing information, facilitating research and education and the potential for the development of novel technologies such as AI.

#### 3.3.1. Increased Efficiency and Technical Aspects

There was a strong perception that adoption of a digital workflow would reduce turnaround time, thus reducing patient anxiety. Some men felt that reviewing digital images would lead to greater diagnostic accuracy, if images were to be of sufficient resolution and viewed on large digital displays.

#### 3.3.2. Formation of a Permanent Record

Points were raised regarding the ability to easily archive and retrieve digital images compared to physical glass slides. Many perceived this to be of benefit if multiple biopsies were carried out on the same patient, thereby allowing the comparison of images. A proportion also saw utility in comparison of biopsy findings and post-operative histopathology.

#### 3.3.3. Sharing of Digital Images

The ability to share images among members of the multidisciplinary team was seen as a strong benefit, and for streamlined referral across teams who may be based in geographically distanced centres. In addition to sharing among healthcare professionals, many saw a positive opportunity for images to be shared with them as patients in the clinic setting. Opportunities were seen for the distribution of images for education of trainee histopathologists. Finally, digitisation of images was seen to allow the generation of a bank of data which would be drawn upon for research purposes paving the way for future advances in the diagnosis and treatment of PCa.

**Figure 3:**
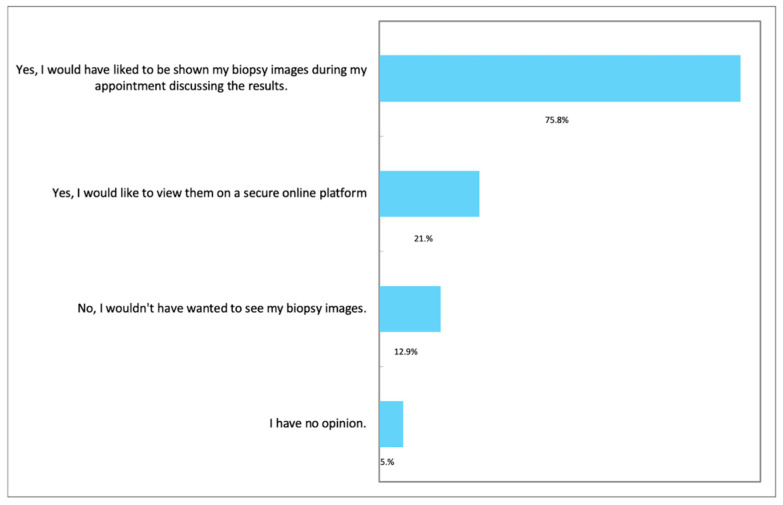
Respondents’ views on viewing digital biopsy images (please see online supplementary material, Question S8). Respondents were able to choose more than one option.

When specifically asked whether they would have been interested in viewing their histopathological images (please see online supplementary material, Question S8, and Figure 3), most men (82.1%, 1048/1276) responded that this would be desirable. More specifically, in terms of the format of this, of those who responded positively, most would prefer this to be in a clinic rather than on a secure online platform.

#### 3.3.4. Deployment of AI Techniques

Many respondents acknowledged the possibility of human error and fatigue in manual reporting of histopathological slides, thus raising the potential advantage of AI as an adjunct to reduce such errors.

Neutral responses suggested indifference to the methods of producing a histopathological report, as long as the report was produced in a timely and accurate manner. Some responses suggested indifference due to personal limitations of knowledge and understanding of possible benefits of the technology.

#### 3.3.5. Reservations towards DP

Few responders were against the adoption of DP were relatively few, but important ideas were raised. Participants discussed the need for digital images and associated data to be held on a secure server, well protected from illicit access and accidental loss. Others felt a greater sense of trust in reports generated purely by a human without any computational intervention or distrust in computer systems.

### 3.4. On the Introduction of AI in the Reporting of Histopathology

**Figure 4:**
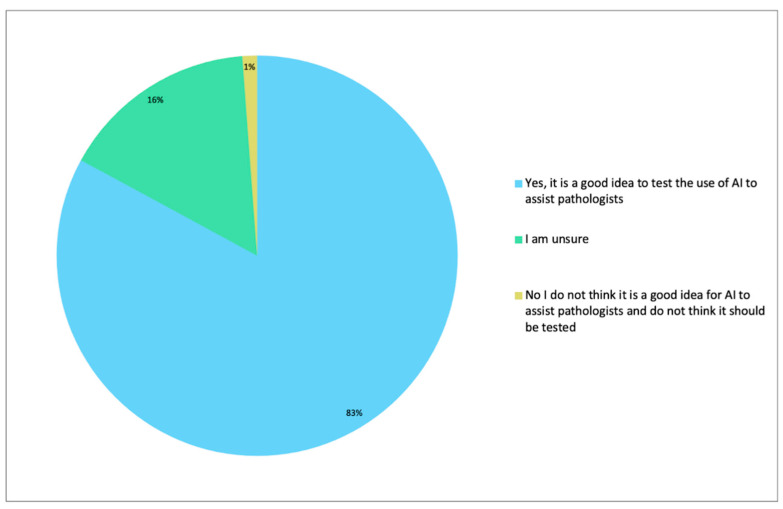
Digital developments could allow Artificial intelligence (AI) to be used in histopathology. Pathology AI means that a computer programme can potentially assist with the diagnosis of prostate cancer by double checking results. To find out for certain, more testing is being carried out. What do you think about research that will test whether pathologists can be assisted by AI when diagnosing prostate cancer?

Although most men (1058/1276, 83%) considered the testing of AI as an adjunct to histopathological reporting (please see online supplementary material, Question S9, and Figure 4) to be a positive advance, some were unsure (203/1276, 16%) and a few (15/1276, 1%) were not in favour.

**Figure 5:**
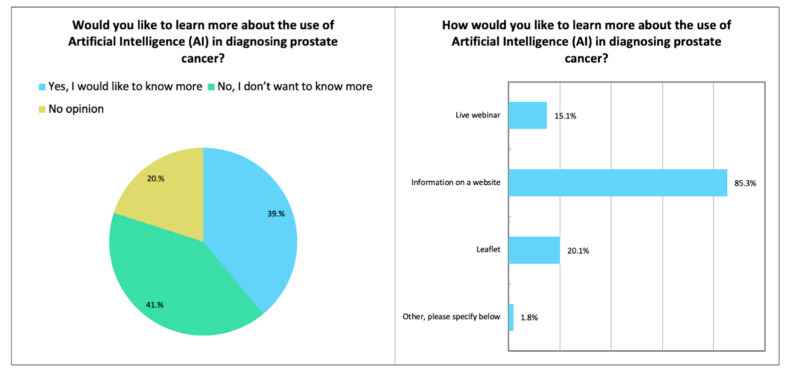
Visual representation of respondents’ views on whether they would like to be further informed about AI in the diagnosis in prostate cancer and the preferred format (please see online supplementary material, Question S10).

A range of views were observed regarding whether patients wished to learn more about the use of AI in the diagnosis of PCa (please see online supplemental material, Question S10, and Figure 5). In total, 39% (498/1276) of respondents wished to learn more, 41% (524/1276) did not and 20% (254/1276) had no opinion. Of the 498 who desired further information, the majority preferred for this to be in website format (85%, 425/498) rather than a leaflet (20%, 100/498) or live webinar (15%, 75/498), and 1% (6/498) were open to receiving information by email.

Most comments were positive (examples in Table 2). Free comments offered by men who were ambivalent or against the use of AI were categorised into two main themes: (1) technical performance; and (2) preference for human review with a greater sense of personal trust versus the output of an algorithm. The 5/15 who were not in favour provided additional free text explanations. Two related to general mistrust of AI, two referred to wanting human input in decision making and one was uncertain about the accuracy. A summary of the free text comments for those who were unsure related to having insufficient knowledge, concerns around accuracy or performance (*n* = 30), wanting human input or double checking by a human (*n* = 18), those who were generally unsure or sceptical (*n* = 9) and those who would defer the decision to use the technology to an expert (histopathologist) (*n* = 6). Others included ensuring resources were not diverted from elsewhere (*n* = 1), no comment (*n* = 1) and not enough knowledge of histopathology to comment (*n* = 1). One respondent made two comments, so these are indicated as two comments, all others made one comment.

#### 3.4.1. Technical Performance

Comments discussed the wish for a reliable result, and concerns raised regarding possible misdiagnosis although some noted that apprehension would be alleviated if more data were available for reassurance regarding the performance of the algorithms.

#### 3.4.2. Preference for Human Review

Respondents expressed reassurance from involvement of a trained professional in the generation of a histopathological report. The expertise of an experienced histopathologist was felt to be preferable to AI in terms of patient confidence in the result. Some in this group stated they would be reassured if the performance of an AI algorithm be verified by a histopathologist rather than function autonomously.

## 4. Discussion

The insights gained from this study demonstrate that the majority of men undergoing a prostate biopsy are supportive of the use of technology in the form of digital pathology or AI for diagnostic assistance in assessing their prostate biopsy.

An advantage of digital pathology is that it offers the ability to make histopathology images more accessible, and a recurring theme amongst the free text comments was around image sharing in various guises: for expert opinion, for teaching and education for clinicians (pathologists) and for patient information. Indeed, the survey has shown that there is an apparent patient desire to review their images alongside a healthcare professional in clinic, which may not be intuitive. Although this practice is commonplace for radiology, surgeons and oncologists are less familiar with histopathology, and this is an obstacle in the adoption of this form of clinical practice. In a recent survey setting in a testicular tumour network, oncologists potentially felt that viewing digital pathology images may complicate discussions, especially if a pathology-related question was asked [10]. There may be a role for AI-annotated images to assist the clinician in highlighting pertinent features to the patient in clinic without the need for a histopathologist to be present. We perhaps need to find ways to facilitate this, for example with histopathologists adding user friendly annotations or labels to images to aid non-histopathologists, but this has time and resource implications in a speciality in which 97% of histopathology laboratories in the UK already report too few staff to meet clinical demand [23].

Examples of conversations which could potentially be enhanced by viewing images include small foci of Gleason Grade Group 1 PCa where patients are often recommended to receive active surveillance (AS). A proportion of men commencing AS may subsequently receive treatment for “non-biological” reasons such as patient anxiety and uncertainty, with rates of 8–23% across different studies [24]. Although untested, if patients could see for themselves that their cancer is small and low grade then this might provide reassurance that it could be managed by AS. Other discussions that may be informative include different Gleason grades that are present or demonstrating that immunohistochemistry has been performed to confirm small areas of cancer for reassurance that the diagnosis is correct. Although highly relevant, a detailed discussion on conveying if and how AI results were used in formulating the histopathologist’s diagnosis to a patient during assessment is beyond the scope of this paper and the experience of the authors to date will form the basis of further studies.

The outputs of this study provide useful insight for anyone looking to deploy AI outside of a retrospective, research setting and into a live clinical diagnostic workflow. We have demonstrated that the majority of men are in favour of testing AI for diagnostic assistance and potential benefits of improving diagnostic accuracy, increasing capacity within histopathology departments and standardising subjective assessments such as Gleason grading, which can be reassuring. We reiterate that the intended utility for AI systems in histopathology is as an adjunct to review by a histopathologist—there is opportunity to better and more clearly communicate this point to the general public. It could be a criticism of this study that in order to avoid overly complex medical terminology the neutrality of the overall tone of some questions may have appeared more in favour of the benefits of DP/AI.

It remains important to explore the views of those who are unsure or do not think this is a good idea to allay concerns where possible. The respondents who were unsure or not in favour of AI assistance had concerns that could potentially be addressed with more information or better communication. For example, in the ‘not in favour’ group, only 2/5 were genuinely distrustful of AI. In the ‘not sure’ group, most comments related to wanting human input, wanting more information about the relative performance of the tool or deferral of the ultimate decision whether to use the technology to an expert (histopathologist). We take away from this that in providing information to patients about AI and histopathology, the key messages that need to be conveyed are that currently these tools are an assist to histopathologists, with those same clinical experts still retaining oversight and responsibility for the diagnostic report. If AI is considered part of clinical care, there will likely be institutional opt out processes for certain parts of clinical care which those with a very strong objection can invoke.

It is important to understand that the adoption of machine-assistance in healthcare is not limited to histopathology. In surgical practice, the robotic-assisted radical prostatectomy was introduced in 2001 [25] and has been associated with favourable outcomes over traditional radical prostatectomy [26]. Men who had experienced such surgery were generally satisfied with their experience, although there was a lack of understanding in some regarding the precise role of the surgeon and the robot in such circumstances [27]. Another more general British study [28] gauged differing viewpoints of male and female patients when faced with robot-assisted surgery. Male participants were generally found to be less concerned about the adoption of the technology although female participants found the use of a robot to be dehumanising. Similar opinions were raised by some respondents in our study although given our focus on PCa patients, our cohort is exclusively male.

Within the diagnostic specialties, perhaps ahead of pathology in the adoption of AI-assisted diagnosis is radiology. A group in the Netherlands [29] explored viewpoints of patients undergoing radiological studies regarding the implementation of AI in reporting radiology. In summary, patients expressed distrust towards AI reporting, wished for preservation of human interaction and were generally ambivalent towards potential workflow efficiency gains. The domains discussed were similar to those in this study; however, a considerable difference is observed regarding the results, with many radiology patients being more reserved regarding the adoption of AI. The reasons for these reservations were comparable those expressed by our cohort of respondents.

Respondents to our survey voiced concerns over data governance relating to digital images, which has recently been identified as a source of concern amongst the histopathology community [30]. Issues regarding data retention, storage, security and use for secondary purposes such as for education and research have been raised [30,31]; however, these issues relate to glass slides also. It is believed that, currently, no specific guidance is in place to govern the application of whole slide images to the research setting in the UK. Guidance on the validation of DP for diagnosis has been issued by the Royal College of Pathologists (RCPath), although issues of data governance currently fall outside the scope of the document. Although not yet formally adopted in the UK, such guidance has been produced by the Canadian Association of Pathologists [32].

Some respondents considered the retention of data within the UK a necessity and voiced concerns against foreign interference. With regard to storage of DP data for clinical purposes, these would be governed by existing principles of patient confidentiality as set out by UK legislation and General Medical Council policy. These would then be normally kept within the UK. Regarding cross-border transfer of information, a framework set out by the Organisation for Economic Co-operation and Development [33] would be the principle by which privacy is maintained.

The adoption of DP and AI into routine practice raises the question of where ultimate responsibility will lie in the event of a clinical error and this has been the subject of some debate [34]. Due to the relative infancy of the technology and its, thus far, limited real-life use, there is no current precedent for this. It may be that, with time and further scrutiny by regulatory bodies, this becomes clearer. Our results demonstrate that patients are more comfortable with the overall responsibility for a histopathology report remaining with the histopathologist and relying on their decision making to use AI and integrate its findings into the final report.

In summary, we have found that most respondents are supportive of advances in PCa diagnosis by means of DP and AI-assisted diagnostics as adjuncts to current workflows. A potential confounder of the responses we have observed is that these have originated from supporters of a prostate cancer charity and may, therefore, reflect a more engaged patient population. There is, however, a small population with reservations for whom trust must be maintained. Patient reassurance that these methods serve to enhance the diagnostic method, rather than replace it, may be the means by which to achieve this. While the results of this study can provide general insight into patient perception of the testing of AI in histopathology, our cohort is limited to men and PCa in the UK. Further work may, then, be of utility exploring the views of other populations. These may include a more global cohort, women or those with other cancers such as breast cancer.

## Figures and Tables

**Table 1:** Example free text quotations from respondents on their views regarding the introduction of DP into routine practice.

Theme	Example Quotations
(a) Efficiency and technical aspects	Having had prostate cancer anything that speeds up and increases the accuracy of diagnosis has to be good. I am no expert but anything that makes viewing samples easier should make the doctors work easier and perhaps more accurate. I have done microscope work before, and it can be tiring and challenging especially if you are trying to count occurrences over an area. Digital leaves less room for human error. It sounds positive but without lots more information I could not say very positive, how secure will this data be, how reliable is the digital screening, is it at least as reliable… as a human?
(b) Record permanence	As a digital record, it can be transferred between departments enabling specialists to discuss it. More importantly, it can’t be lost easily. A permanent record would provide a baseline assessment in case of need for further biopsies. A digital record could be kept for MDT meetings. I approve of the positive digital record, but the sample(s) should not be discarded before a conclusive diagnosis (higher magnification may be needed than the digital images). Any use of more modern technology cannot be a negative as long as the control over data is maintained properly/adequately. I suppose I wouldn’t like my digital records getting into the wrong hands!!
(c) Sharing of images	A digital record is easily stored and can be easily shared with appropriate people. If requested, it could be shared with the patient to aid understanding of the result. Ability to share between experts, and ability to share/show the patient. There is a digital record of the sample(s) which can easily be recalled as a basis for comparison if there is/are ever repeat tests. Also, the record will be an element in a database of all biopsies which might be valuable for statistical or other test purposes. Having an electronic library enables medical staff and researchers to have better access. I had three biopsies in all… I trusted the histopathologists to produce the necessary reports, which were then used to decide the way ahead. The first biopsy was abroad, and I actually brought the original slides back to UK with the report; that would, of course, have been easier had they been digital. As a patient I have no particular view on digital vs. analogue slides, except that digital probably eases record-keeping and referral. I would have been very interested to have seen the samples and had their significance explained to me. Makes the data/information available for study/research/analysis to many more specialists instantly. My consultant explained in detail what the outcome was from the histology, but I did not see a digital scan of the biopsies. I think that would be helpful. Opportunity for referring back to the images. Allows teams to see the images and comment. Other centres able to easily review the histopathology ensuring uniformity in research, etc. This should enable retrospective scanning back over images if something useful is discovered in the future, where historic biopsy data, perhaps combined with progression/survival data, would be useful. However, it’s important the biological samples are retained too, so they can be used for things like genomic sequencing.
(d) AI	AI is infallible if programmed correctly As long as a person who checks the results is experienced in reading them and the digital image is not a just compared to a digital library. Digital images can be stored to compare at a later date, it should be a positive move. Assuming the quality of image is as good, and the data is properly managed, the added availability should help diagnosis. It may also assist machine learning for analysis and more accurate diagnosis. It seems to make sense to digitize records. It should speed their transfer from one party to another and will make it easier for more than one professional to examine them. It might also facilitate the use of ai to review them. Provided the “library” of samples was large quantities and good enough quality, using automated digital imaging can cover more areas of the “slide” and present the targeted cells for review by qualified human to maximise efficiency and throughput. AI technology can help and be taught to find needles in haystacks… Can machines interpret as well as humans in this situation, I don’t know the answer to this question?
(e) Reservations towards DP	The important factor for me as a potential cancer sufferer was to know that every process was being done to the best level possible. I did not want or need to know the details of the process itself but trusted that I was getting the best. As techniques advance, I think I would have the same attitude. Trust that the people know what they are doing and using the best technology. Either cancer is present or its not, how you record it is immaterial. I am only interested in the results. As a patient I am just interested in the results regardless of how they have been arrived at. I would think that an expert examining the sample would always be more reliable than a computer, but maybe there is a role for both approaches if there is time and money available Is it necessary to keep digital records? Once you know the result, a record is kept. So what? How is this going to help me?

Footnote: please also see online supplementary material—Question S7.

**Table 2:** Example free text quotations from respondents on their views regarding the introduction of AI into routine histopathology reporting.

Theme	Example Quotations
(a) Support for AI	AI might pick up things a tired histopathologist missed, so having them confirm each other’s work would be good. I think evidence shows that the same histopathologist analysing the same slide sometime later does sometimes give a different grade. Getting the grading right is important for picking the right treatment. As long as AI does not become the primary decision maker but takes the strain on some of the more mundane elements of the process, I wouldn’t have a problem. Assuming that AI can meet (maybe exceed) the levels of accuracy of a human this could free up the experts’ time for other uses. Clearly research should be carried out to see whether AI could help. But it should only be pursued if rigorous checking indicates there are benefits over and above what a pathologist can do. I think AI makes sense as long as its role is to assist and not to take over from a trained pathologist. I wouldn’t be comfortable with the latter at this stage, but I see value in perhaps helping to increase the speed of diagnoses, add a degree of consistency in diagnoses which can sometimes be difficult for a pathologist to achieve all the time, and possibly to help detect patterns across a number of patients leading to potential future research and treatment areas. I think it is a good idea; however, a senior pathologist needs to verify the results. Possibly the most important element to modern AI/pattern recognition methodologies is a good reliable data set and to not over train the network. It is essential, therefore, to ensure the quality of the ‘AI’ and also to ensure that if samples are not confirmed by human examination, then the system produces zero false negatives. Also, it is essential to be very cautious of using some ‘AI’ companies as partners as they are nothing short of charlatans. AI has a great potential to speed up diagnosis, a benefit to patients. Quality control would be essential, checking that known true positives are picked up, and regular sampling so a histopathologist can check for false positives and false negatives. My only concern is that it may eventually lead to fewer pathologists being employed and by them becoming “de-skilled”.
(b) Concerns regarding technical performance	I understand that AI is cost effective and probably can get through a greater workload quicker. My concern, again, is that will something be missed if the programming or the technical quality of the ai is compromised. Good idea but the usual checks and balances need to be good and regularly tested—see post office debacle re their post masters and mistresses.
(c) Preference for human review	I’m all for the advancement of technology, but for the use of diagnostic purpose I would prefer the opinion of a doctor. You can’t program experience and a “hunch”. As long as AI is used alongside pathologist looking at the data, all will be good but should not replace a pathologist. AI may be something that a younger generation accept without question but for an old dinosaur like me AI Is far from second nature. This does not mean that it couldn’t be useful, it just means that I need convincing. As a lay person, I would have thought it would assist professionals, but I would not want it to be totally replacing an expert.

Footnote: please also see the online supplementary material—Question S9.

## Data Availability

Raw data available on request.

## Supplementary Materials

The following supporting information can be downloaded at: https://www.mdpi.com/article/10.3390/diagnostics12051225/s1. Please see Survey Questions.
