# Supplementary material for: The Use of Digital Pathology and Artificial Intelligence in Histopathological Diagnostic Assessment of Prostate Cancer: A Survey of Prostate Cancer UK Supporters
Source: Diagnostics (Basel). 2022 May 13;12(5):1225. doi: 10.3390/diagnostics12051225 (PMC9141178; doi:10.3390/diagnostics12051225)
Supplement: Supplementary file 1 [file diagnostics-12-01225-s001.zip › diagnostics-1700768-supplementary.pdf]

SUPPLEMENTAL MATERIAL (ONLINE PUBLICATION ONLY):

SURVEY QUESTIONS

Introductory statement:

Thanks for taking the time to complete this survey for Prostate Cancer UK. We are working with Oxford University to speak to men who have had a prostate biopsy to find out your experience and understanding of this. Your feedback will help practitioners improve the biopsy process and inform future developments in this field. The results may also be published in an academic journal to help others understand patients' thoughts and worries.

The survey has 11 questions and should take about 10 minutes to complete. All answers are anonymous and will only be used for the purposes of this research. For questions which you may find sensitive, we have provided a 'Prefer not to say' option.

1. **Have you had a prostate biopsy in the past?**
   - Yes
   - No
   - Don't know
   - Prefer not to say

If answered 'no' or 'don't know', the following message was displayed:

Thank you very much for taking the time to take part. Unfortunately, you do not fit our criteria for continuing with the survey.

If you would like more information on prostate cancer, please visit: https://prostatecanceruk.org/

Whether you've been diagnosed or have concerns about prostate cancer, Prostate Cancer UK are here to support you. You can call our Specialist Nurses for information or support on 0800 074 8383. They'll listen to your worries and questions in confidence. They can also give you information about other services that may be useful.

2. **When did you have your biopsy?**
   - Before 2017
   - In 2017 or after
   - I'm not sure
   - Prefer not to say

3. **If you have had a biopsy taken from your prostate, a histopathology (cellular pathology) department will check for cancer cells and report on how many biopsy samples contained cancer, how much cancer was found in each sample and how aggressive the cancer appears. Before taking this survey, how well did you understand what happens in a histopathology department?**
   - I understood completely what happened in a histopathology department
   - I had heard of a histopathology department but don't understand what they do
   - I had never heard of a histopathology department and didn't know what they did before this survey
   - I am not sure

4. **Doctors who work in histopathology departments specialise in studying cells and tissue under a microscope. These doctors are called histopathologists. Before taking this survey, did you know histopathologists are involved in the diagnosis of prostate cancer?**
   - I know what a histopathologist is and what they did
   - I have heard of a histopathologist but I don't know what they did
   - I have never heard of a histopathologist before this survey
   - I am not sure

5. **Would you like to know more about what histopathologists do in a cellular pathology department?**
   - Yes, I would like to know more
   - No, I don't want to know more
   - No opinion

6. **Please select how you would like to learn more:**
   - Live webinar
   - Information on a website
   - Leaflet
   - Other

Please explain your answer [free text] or prefer not to answer

7. Some histopathology departments are now going 'digital'. Slides containing prostate biopsy tissue can now be scanned and viewed digitally on a screen rather than through a microscope. This makes a permanent digital record of the biopsy which reduces the chances of any issues with viewing slides. This also allows histopathologists to easily get a second opinion on a diagnosis. Do you see this change in diagnosing prostate cancer as a positive or negative?

- Very positive
- Positive
- Neutral
- Negative
- Very negative
- Not sure

Please explain your answer [free text] or prefer not to say

8. Digital pathology can allow you to view your biopsy images. Is this something you would have liked the chance to do? Please select all that apply:

- Yes, I would have liked to be shown my biopsy images during my appointment discussing the results.
- Yes, I would like to view them on a secure online platform
- No, I wouldn't have wanted to see my biopsy images.
- I have no opinion.

Please provide more details here if you wish [free text] or prefer not to answer

9. Digital developments could allow Artificial intelligence (AI) to be used in histopathology. Pathology AI means that a computer programme can potentially assist with the diagnosis of prostate cancer by double checking results. To find out for certain, more testing is being carried out. What do you think about research that will test whether pathologists can be assisted by AI when diagnosing prostate cancer?

- Yes, it is a good idea to test the use of AI to assist pathologists
- I am unsure
- No I do not think it is a good idea for AI to assist pathologists and do not think it should be tested

Please explain your answer [free text] or prefer not to answer

10. Would you like to learn more about the use of Artificial Intelligence (AI) in diagnosing prostate cancer?

- Yes, I would like to know more
- No, I don't want to know more
- No opinion

11. Please select how you would like to learn more:

- Live webinar

- Information on a website
- Leaflet
- Other, please specify below

If you answered "other", please explain below [free text] or prefer not to answer

At the end of the survey, the following message was displayed:

Thank you very much for taking the time in completing this survey. Your opinions and answers are very much appreciated.

Whether you've been diagnosed or have concerns about prostate cancer, Prostate Cancer UK are here to support you. You can call our Specialist Nurses for information or support on 0800 074 8383
